# Supplementary material for: Structural-functional analysis of engineered protein-nanoparticle assemblies using graphene microelectrodes
Source: Chem Sci. 2017 Jun 13;8(8):5329–34. doi: 10.1039/c7sc01565h (PMC5607901; doi:10.1039/c7sc01565h)
Supplement: Supplementary file 1 [file SC-008-C7SC01565H-s001.pdf]

Electronic Supplementary Material (ESI) for Chemical Science.
This journal is © The Royal Society of Chemistry 2017

Supplementary Material for

Structural-Functional Analysis of Engineered Protein-Nanoparticle Assemblies Using Graphene Microelectrodes

Jinglei Ping[1], *Katherine W. Pulsipher*[2], *Ramya Vishnubhotla*[1], *Jose A. Villegas*[2], *Tacey L. Hicks*[2], *Stephanie Honig*[2], *Jeffery G. Saven*[2], *Ivan J. Dmochowski*[2], *A. T. C. Johnson*[1]

[1]Department of Physics and Astronomy, University of Pennsylvania, Philadelphia, PA 19104 USA

[2]Department of Chemistry, University of Pennsylvania, Philadelphia, PA 19104 USA

Contents

1. Ionic-strength dependence of the assembly state of AfFtn and AfFtn-R

2. Analysis of current response to AfFtn and AfFtn-R.

3. Charge transfer for AfFtn-AA-AuNP, AfFtn-AuNP, and AuNP.

4. Confirmation of AuNP-enclosure

5. AuNP stability in PBS

6. AfFtn and AfFtn-AA 4-nitrophenol Reduction

7. General protein characterization

8. I-BODIPY Characterization

1. Ionic-strength dependence of the assembly state of AfFtn and AfFtn-R

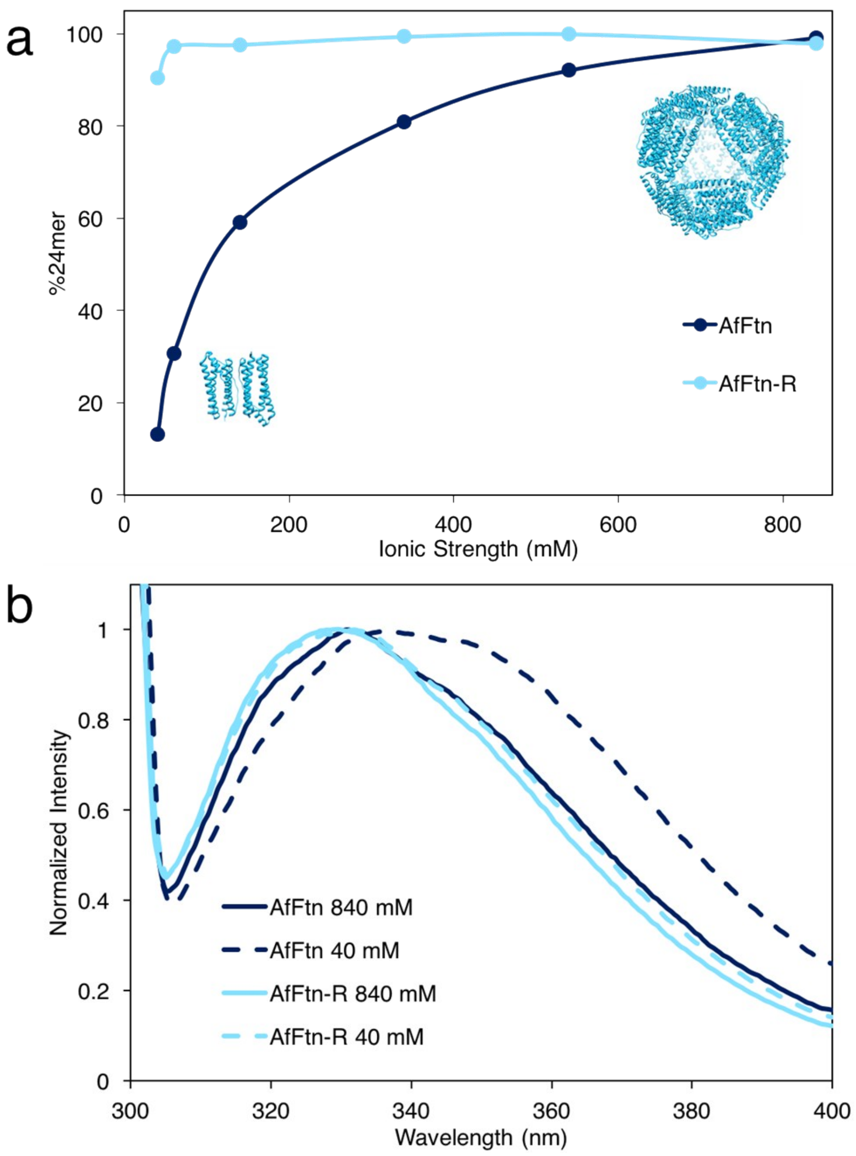

Figure S1. a Size exclusion chromatography was used to quantify the amount of 24mer at each ionic strength (40, 60, 140, 340, 540, 840 mM) by calculating the area under the peaks corresponding to 24mer (elution volume ~10 mL) and dimer (elution volume ~15 mL). Protein solutions were made up at 5 mg/mL at varying ionic strengths and equilibrated overnight at 4 °C. A Superdex200 Increase 10/300 GL column was used on an AKTA FPLC. **b** Tryptophan fluorescence spectra for AfFtn and AfFtn-R in phosphate

buffer solutions of either 40 mM or 840 mM ionic strength. A red shift in emission is indicative of increased solvent accessibility of Trp residues and can be used as a marker for disassembly. AfFtn shows a red shift of ~7 nm in lower ionic strength solution, while AfFtn-R shows essentially no change. Samples are 5 mg/mL and were equilibrated overnight at 4 °C. For fluorescence measurement: excitation wavelength = 295 nm, T = 25 °C, PMT voltage = 800 V.

2. Analysis of current response to AfFtn and AfFtn-R

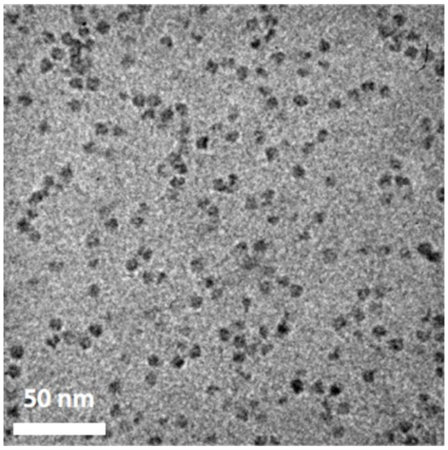

Figure S2. TEM image of ferritin non-specifically adsorbed on graphene. The light gray background is a sheet of monolayer graphene. Dark dots are individual ferritin cages.

The Grahame equation[1] can be written as:

$$\psi_f = \frac{2k_{\mathrm{B}}T}{e}\sinh^{-1}\left(\frac{\sigma_s}{8\varepsilon\varepsilon_0 k_{\mathrm{B}}Tc}\right) \tag{1S}$$

where k_B is Boltzmann's constant, T the absolute temperature, e the electronic charge, ε (ε_0) the relative (vacuum) permittivity, and σ_s the areal charge density in the solution above the graphene.

The data in Fig. 2b are well fit by Eqn. (1S). The best fit values for $R_{\mathrm{ct,24mer}}$ and $\sigma_{\mathrm{d,24mer}}$ are 232 ± 74 GΩ and -0.07 ± 0.03 C m^{-2}, respectively. Compared to the fit values obtained for an experiment conducted in pure phosphate buffer (R_{ct}= 65 ± 3 GΩ, σ_{d}=-0.031 ± 0.003 C m^{-2}), there is an increase of the negative charge areal density due to the negative charge carried by AfFtn-R, accompanied by an increase in the charge transfer resistance due to the inhibition of charge communication by charged AfFtn-R molecules[2,3]. Given the measured areal number density of non-specifically adsorbed ferritin on graphene, ~3500 μm^{-2} (See Fig. S2d), the effective negative charge carried by

each AfFtn-R 24mer is ~40, smaller than the value estimated by the pK_a values of the residues of AfFtn-R, ~110. This is not surprising as charges on ferritin far from the graphene should be screened by ions in solution[4].

The Faradaic current measured for AfFtn solution differs significantly from that for AfFtn-R solution. Unlike AfFtn-R, which remains assembled across the range of ionic strengths tested, AfFtn disassembles into dimers at low ionic strength with a portion of $\frac{K/c}{1+K/c}$, where $K = [NaCl]_{1/2}$ is the salt concentration where the AfFtn is half disassembled[5]. The inverse of the charge transfer resistance $1/R_{\mathrm{ct}}$ and the surface charge density σ_{s} for the graphene/AfFtn solution interface are the sum of these quantities for the dimers, $1/R_{\mathrm{ct,dimer}}$ and $\sigma_{\mathrm{s,dimer}}$, and those for the 24-mers, $1/R_{\mathrm{ct,24mer}}$ and $\sigma_{\mathrm{s,24mer}}$, weighted by $\frac{K/c}{1+K/c}$ and $\frac{1/c}{1+K/c}$, respectively. The current difference can then be written as

$$\Delta i = \frac{2k_{\mathrm{B}}T}{eR_{\mathrm{ct}}}\sinh^{-1}\left(\frac{\sigma_{\mathrm{s}}}{8\varepsilon\varepsilon_0 k_{\mathrm{B}}Tc}\right) - \frac{2k_{\mathrm{B}}T}{eR_{\mathrm{ct,24mer}}}\sinh^{-1}\left(\frac{\sigma_{\mathrm{s,24mer}}}{8\varepsilon\varepsilon_0 k_{\mathrm{B}}Tc}\right) \qquad (2S)$$

The measured current for the AfFtn-R solution is well fit by Eqn. (1S) with best fit values of R_{ct} (232 ± 74 GΩ) and σ_s (-0.07 ± 0.03 C m^{-2}), consistent with our earlier report on graphene microelectrodes in buffer.[6] The Faradaic current difference between the solutions of AfFtn and AfFtn-R (Fig. 2c in the main text) can be explained using the above model. The best fit value for K = 210 ± 60 mM derived from this model is in excellent agreement with the value obtained by liquid chromatography[5] (K = 200 mM). For characterizing the ferritin areal density, graphene was soaked in horse spleen ferritin for 10 minutes, quickly dried by compressed nitrogen air, and characterized by

transmission electron microscopy (TEM). The number density of non-specifically adsorbed ferritin is ~3500 μm^{-2} as shown in the TEM image Fig. S2d.

3. Charge transfer for AfFtn-AA-AuNP, AfFtn-AuNP, and AuNP

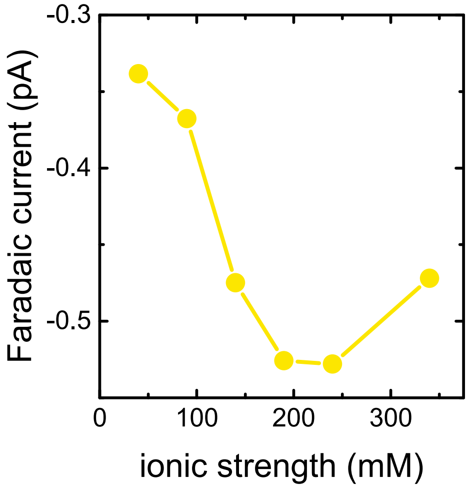

Figure. S3. Faradaic current for 20 nM AuNP as a function of ionic strength by graphene electrodes. The Faradaic current is negative and reaches its maximum magnitude for ionic strength ~240 mM due to competing effects of AuNP-adsorption and ionic screening[7,8].

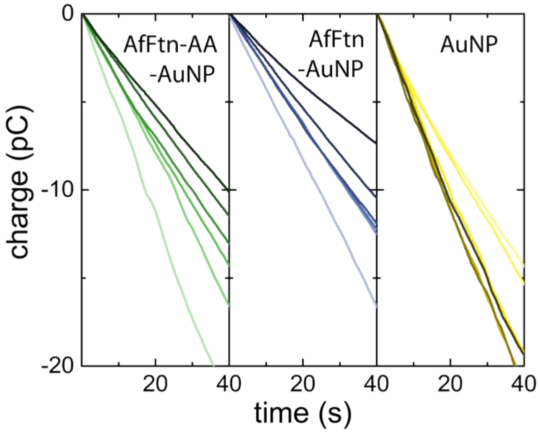

Figure S4. Real-time charge transfer for AfFtn-AA-AuNP, AfFtn-AuNP, and AuNP measured by graphene electrodes. All solutions are at 20 nM concentration.

4. Confirmation of AuNP-enclosure

There are very few free AuNPs in the AfFtn-AuNP solution and AfFtn-AA-AuNP solution. Almost all AuNPs (>99%), as shown in the TEM images of Fig. S5, are enclosed by ferritin 24-mers. We performed this analysis on 12 TEM images, each with approximately 50 ferritin-AuNP complexes. Zero bare AuNPs were found in these images, so we believe that the estimate of 99% encapsulation is conservative.

We note that microelectrode measurements were performed on solutions of AuNP (Supp. Fig. S3), AuNP-AfFtn, and AuNP-AfFtn-AA (Fig. 3a), all at the same concentration of 20 nM. The current variation for (open pore) AfFtn suggests efficient transport through the AuNPs (60%), while that for (closed pore) AfFtn-AA shows essentially zero transport through AuNPs. Assuming that current via bare AuNPs scales with concentration, these observations are fully consistent with our estimate of < 1% free AuNPs.

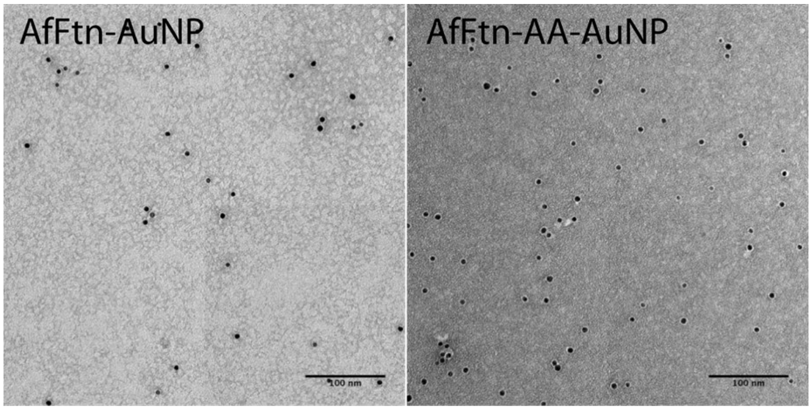

Figure S5. The TEM images for AfFtn-AuNP solution and AfFtn-AA-AuNP solution. The black dots are AuNPs. >99% are enclosed by a white, halo-like ring, which corresponds to the protein shell.

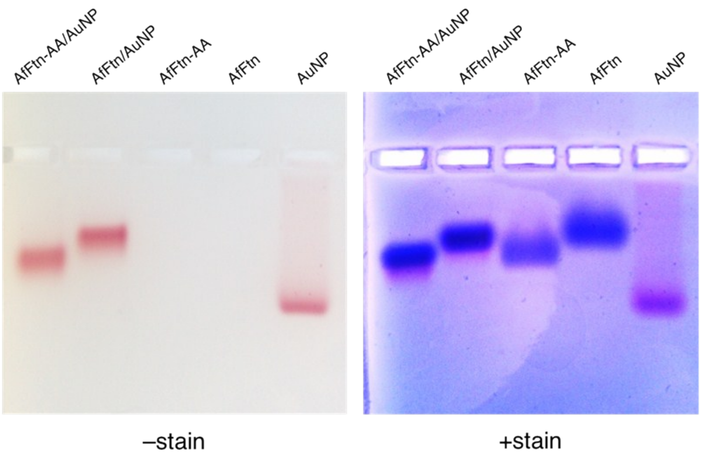

Figure S6. Native gel electrophoresis also confirms encapsulation. Gel was run at 100 V for 20 min. Overlapping red AuNP bands and blue protein bands (+stain gel) indicate encapsulation.

5. AuNP stability in PBS

To confirm that the AuNPs do not aggregate in PBS with ionic strength ranging from 40 mM to 340 mM, surface plasmon resonance (SPR) measurements were performed. As shown in Fig. S6, the maximum of the SPR peak for each value of ionic strength lies in a small range 520.8 ± 1.1 nm, and does not show any obvious trend.

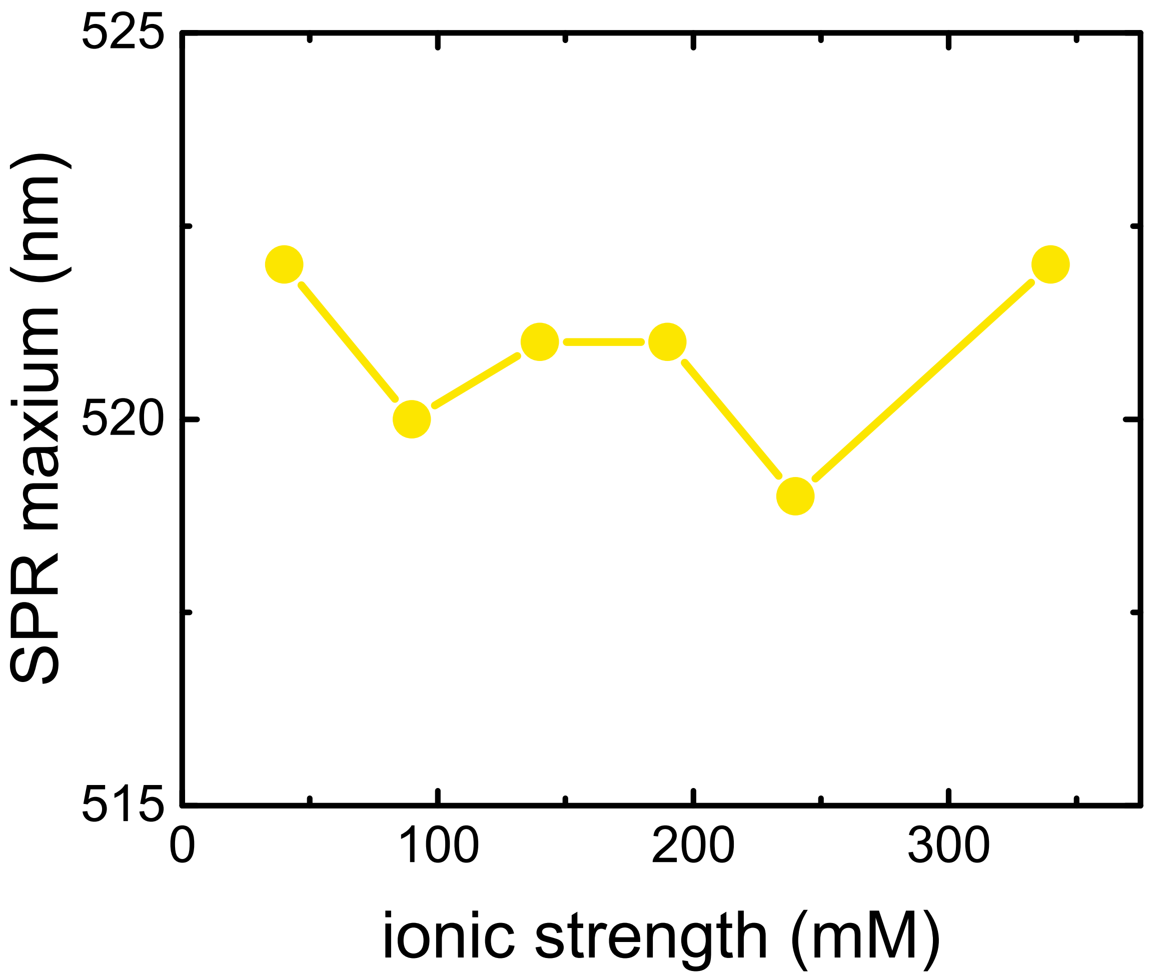

Figure S7. Maximum of the surface plasmon resonance peaks measured for gold-nanoparticle PBS solutions with ionic strength ranging from 40 mM to 340 mM.

6. AfFtn and AfFtn-AA 4-nitrophenol Reduction

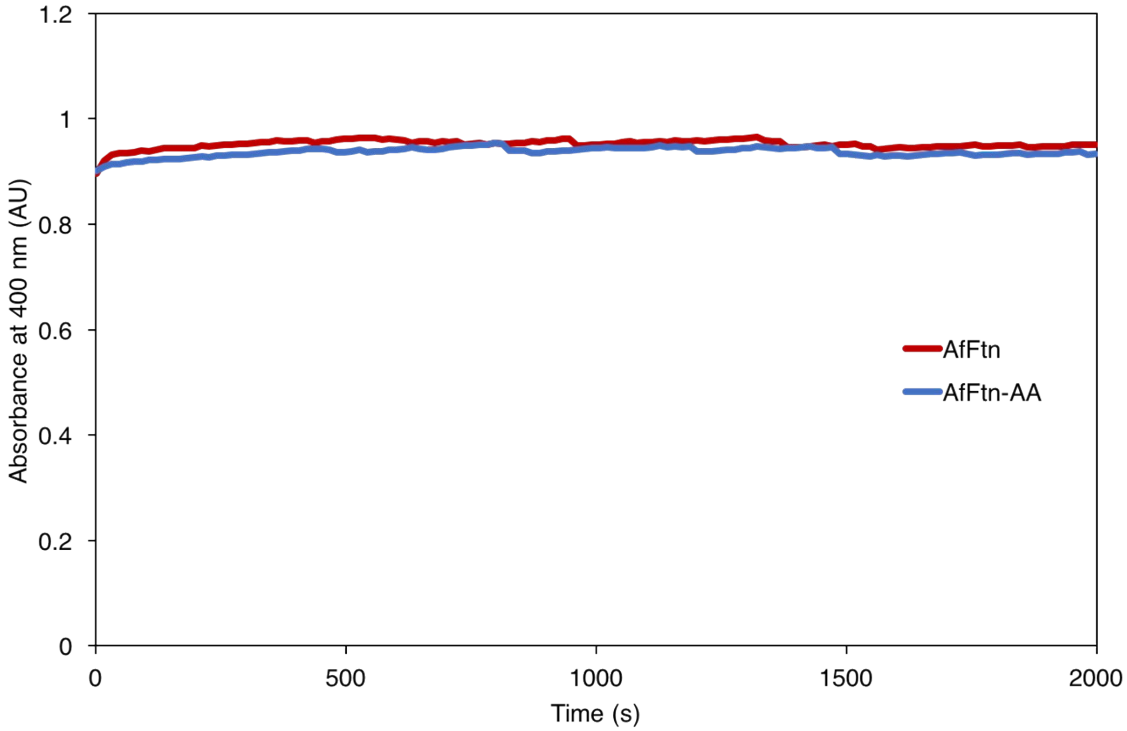

Figure S8. Reduction of 4-nitrophenol in the presence of AfFtn (red) or AfFtn-AA (blue). Neither protein shows any change in A_{400}, indicating no catalytic activity.

7. General protein characterization.

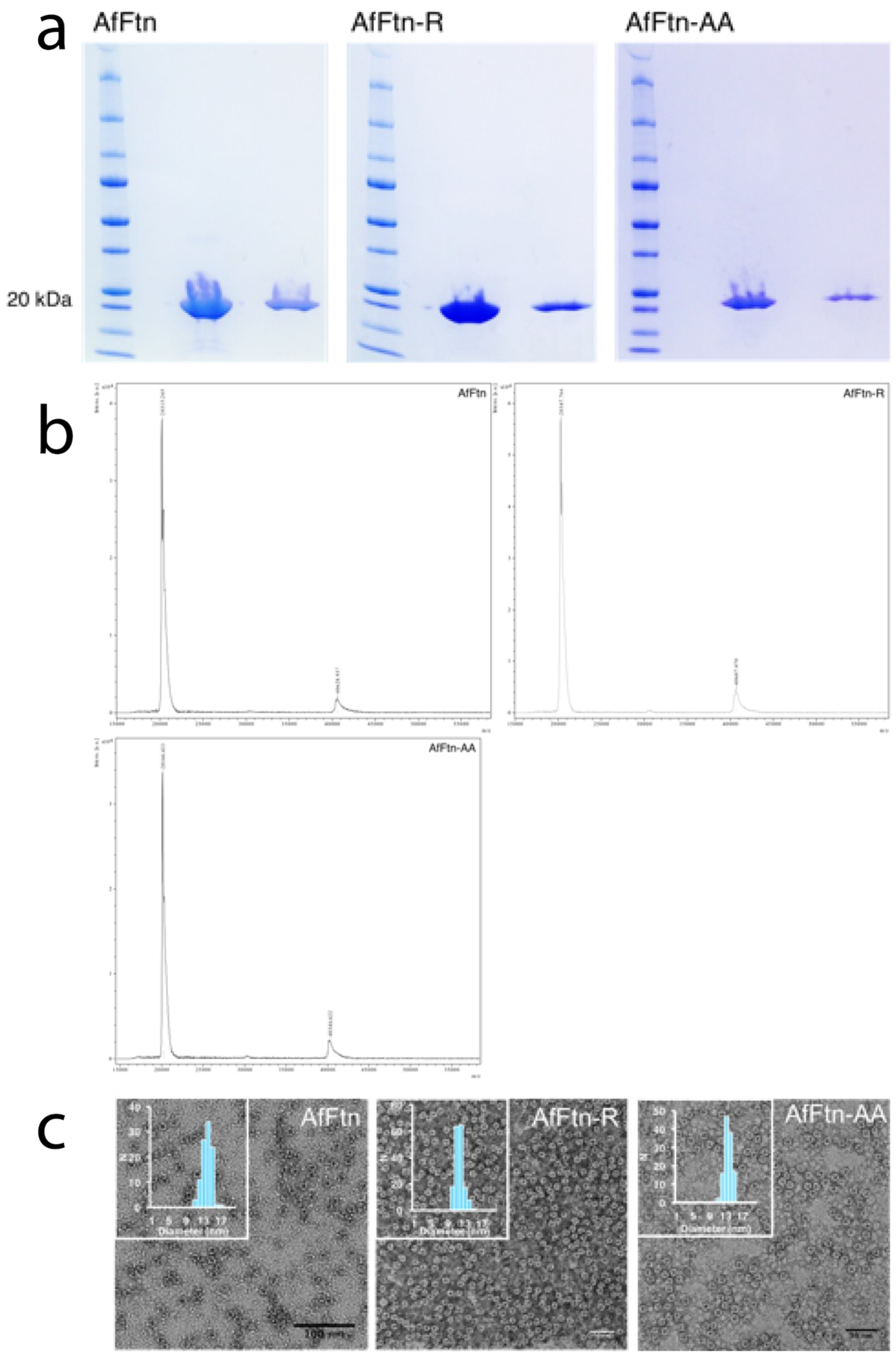

Figure S9. a Denaturing PAGE shows pure protein for all three samples, with monomer MW approximately 20 kDa, as expected. **b** MALDI-TOF-MS for all proteins. Measured

mass for monomer (expected): AfFtn 20315.3 (20316.1 Da), AfFtn-R 20347.8 (20343.1 Da), AfFtn-AA 20166.3 (20173.9 Da). Peaks at ~40 kDa correspond to dimer. Sinapinic acid was used as a matrix, and linear-positive mode was used as the method. **c** TEM micrographs of all proteins under high ionic strength conditions (840 mM). Fully assembled cages are visible for all samples. Samples were prepared on carbon-coated copper grids and were stained with either 2% uranyl acetate or 2% ammonium molybdate. A Tecnai T-12 microscope was used, operating at 120 keV. Scale bars are 100 nm for AfFtn, and 50 nm for AfFtn-R and AfFtn-AA. Particle size was measured manually using ImageJ (NIH).

Table S1. Ferritin Cage Size Summary

**Sample**	**TEM Diameter (nm)**	**DLS Diameter (nm)**
AfFtn	13.2 ± 1.1	13.5 [0.035]
AfFtn-R	11.1 ± 0.9	12.9 [0.059]
AfFtn-AA	12.9 ± 0.9	13.8 [0.082]

8. I-BODIPY Characterization

a

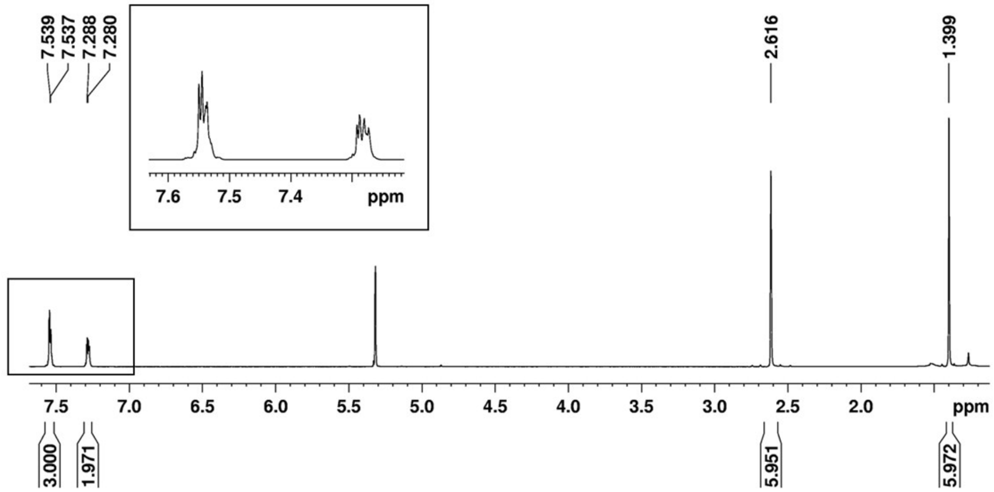

b

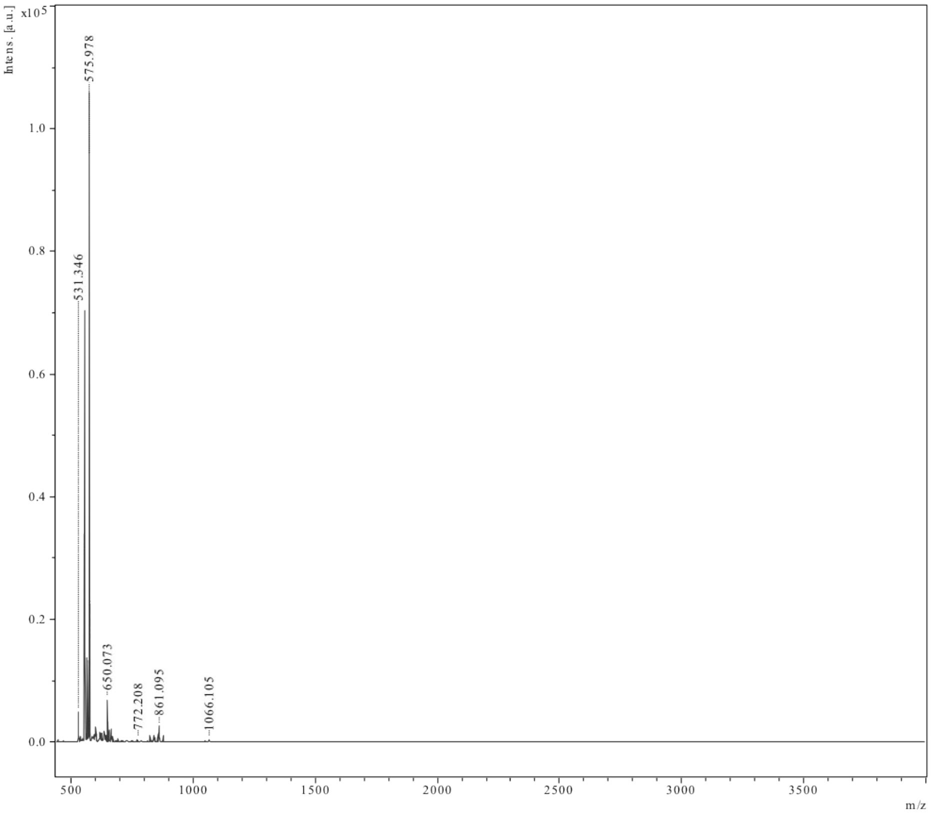

Figure S10. A) ^{1}H NMR spectrum of I-BODIPY in CD_2Cl_2. B) MALDI-TOF-MS spectrum of I-BODIPY using CHCA as matrix. Peaks at 531.346, 650.073, 772.208, 861.095, 1066.105 m/z correspond to matrix. Observed sample mass is 575.978, expected is 575.954.

References

1 Israelachvili, J. *Intermolecular and surface forces: revised third edition*. (2011).

2 Wang, C. H., Yang, C., Song, Y. Y., Gao, W. & Xia, X. H. Adsorption and direct electron transfer from hemoglobin into a three-dimensionally ordered macroporous gold film. *Advanced Funcional Materials* **15**, 1267-1275 (2005).

3 Kim, B. S., Hayes, R. A. & Ralston, J. The adsorption of anionic naphthalene derivatives at the graphite-aqueous solution interface. *Carbon* **33**, 25-34 (1995).

4 Ping, J., Xi, J., Saven, J. G., Liu, R. & Johnson, A. T. C. Quantifying the effect of ionic screening on protein-decorated graphene transistors. *Biosensors and Bioelectronics*, doi:10.1016/j.bios.2015.11.052 (2015).

5 Swift, J., Butts, C. A., Cheung-Lau, J., Yerubandi, V. & Dmochowski, I. J. Efficient self-assembly of Archaeoglobus fulgidus ferritin around metallic cores. *Langmuir* **25**, 5219-5225, doi:10.1021/la8040743 (2009).

6 Ping, J. & Johnson, A. T. C. Quantiying the Intrinsic Surface Charge Density and Charge-Transfer Resistance of the Graphene-Solution Interface through Bias-Free Low-Level Charge Measurement. *Applied Physics Letters* **109**, 013103 (2016).

7 Rance, G. A. & Khlobystov, A. N. Nanoparticle-nanotube electrostatic interactions in solution: the effect of pH and ionic strength. *Physical Chemistry Chemical Physics* **12**, 10075-10780 (2010).

8 Ekiz, F., Yuksel, M., Balan, A., Timur, S. & Toppare, L. Electrochemical polymerization of (2-dodecyl-4,7-di(thiophen-2-yl)-2H-benzo[d][1,2,3] triazole): A novel matrix for biomolecule immobilization. *Macromolecular Bioscience* **10**, 1557-1565 (2010).
